# Expression of Derlin-1 and its effect on expression of autophagy marker genes under endoplasmic reticulum stress in lung cancer cells

**DOI:** 10.1186/1475-2867-14-50

**Published:** 2014-06-11

**Authors:** Li Xu, Zan-Hong Wang, Dong Xu, Gang Lin, Dai-Rong Li, Tao Wan, Shu-Liang Guo

**Affiliations:** 1 Department of Respiratory Medicine, The First Affiliated Hospital of Chongqing Medical University, Chongqing 400016, P.R. China; 2 Department of Obstetrics and Gynecology, The Shanxi Da Yi Hospital, Taiyuan 030001, P.R. China; 3 Center for Disease Control and Prevention, Gaoxin/Xinshi, District of Urumqi, Xinjiang Province, P.R. China

**Keywords:** Derlin-1, Autophagy, ER stress, p62

## Abstract

**Background:**

Recent findings indicated that Derlin-1 has an important function in tumour progression. In this study, we aimed to determine whether Derlin-1 has an oncogene function as a cross-talk molecule with autophagy.

**Methods:**

Cancer cells were treated with tunicamycin (TM) for 8 and 24 h. The expression of Derlin-1 and autophagy-related genes was determined by western blot. Autophagy was analysed by fluorescence microscopy after staining the cancer cells with monodansylcadaverine. The interaction between Derlin-1 and other proteins was identified using co-immunoprecipitation assay.

**Results:**

Our study demonstrated high Derlin-1 expression levels in most non-small lung cancer cell lines. Derlin-1 expression was enhanced under endoplasmic reticulum (ER) stress. Previous studies revealed that TM triggers the initiation of autophagy by activating Beclin 1, converting LC3I to LC3II and degrading p62. Knockdown of Derlin-1 did not affect Beclin 1 and LC3II expression but disrupted the degradation of p62 under ER stress, which resulted in the blockage of autophagy flux. Furthermore, Derlin-1 and p62 were observed to interact under ER stress.

**Conclusion:**

This study is the first report about the interaction between Derlin-1 and p62. Derlin-1 may function in tumour progression partially by interacting with p62.

## Background

Hypoxia and low sugar microenvironment can induce the growth of malignant tumours. Cancer treatment can induce the endoplasmic reticulum (ER) stress response, and lead to misfolded or unfolded protein aggregation in the ER lumen and intracellular Ca^2+^ balance disorders. Unfolded or misfolded proteins need to be degraded in the ER to maintain homeostasis. Protein degradation has been established as a major effector that governs the levels of individual proteins. Protein degradation currently has two main pathways, namely, the ubiquitin-proteasome-mediated and autophagy-lysosome-mediated degradation pathways. For many years, researchers believed that the ubiquitin–proteasome system (UPS) and autophagy function independently. They also believed that the UPS and autophagy are fulfilled by distinct molecular effectors, separate subcellularly and act on mutually exclusive substrates. However, recent findings strongly suggested the existence of crosstalk and cooperation between these two degradation pathways [1,2]. Researchers have identified several active molecules between the UPS and autophagy in both normal and abnormal cells [3-5].

Derlin-1 was originally identified as a US11-interacting protein required for cytomegalovirus-mediated turnover of the class I major histocompatibility complex (MHC) heavy chain 1, as well as an ER membrane-binding site for p97/VCP [6]. The p97 ATPase [also called valosin-containing protein (VCP) or Cdc48 in yeast] and its associated co-factors, Ufd1 and Npl4, interact with VCP-interacting membrane protein, Derlin-1 and poly-ubiquitinated protein to facilitate protein retrotranslocation from the ER lumen to the cytoplasm for degradation by 26S proteasome [6-10]. Recent studies reported that Derlin-1 is overexpressed in carcinomas, such as lung cancer, breast cancer and colon cancer [11-13]. Derlin-1 knockdown was also reported to sensitise breast cancer cells to ER stress-induced apoptosis. These findings demonstrated the potential of Derlin-1 as a new oncogene. The definite molecular mechanisms underlying the retrotranslocation of ER proteins into an oncogene need to be clarified. An increasing body of data showed the link between ER function and autophagy. However, little is known about the relationship between Derlin-1 and autophagy genes. In this study, we explored the effect of Derlin-1 on autophagy-related genes under ER stress in lung cancer cells. Our results show that Derlin-1 knockdown could impair both p62/SQSTM1 (p62) degradation and the interaction between these two molecules. Further investigation is needed to provide additional insight into the potential of Derlin-1 as a therapeutic target.

## Results

### Derlin-1 expression in lung cancer cell lines

**Figure 1:**
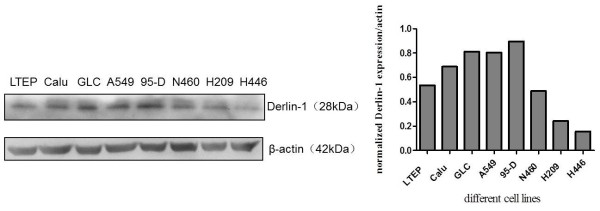
**Detection of Derlin-1 expression in a panel of lung cancer cells by western blot.** Derlin-1 expression was detected in all cell lines. Derlin-1 expression was high in 95-D, A549, Calu-3, GLC-82, NCI-N460 and LTEP-a-2, but low in NCI-H209 and NCI-H446.

We detected Derlin-1 expression in various lung cancer cell lines, namely, adenocarcinoma cell line A549, LTEP-a-2, Calu-3, GLC-82, large cell lung cancer cell line NCI-N460, small lung cancer cell line NCI-H209, NCI-H446 and highly metastatic lung cancer cell line 95-D. Results show that Derlin-1 expression could be detected in all the tested cell lines (Figure 1). Higher levels of Derlin-1 expression were detected in 95-D, A549, Calu-3, GLC-82, NCI-N460 and LTEP-a-2, whereas lower levels of Derlin-1 expression were detected in NCI-H209 and NCI-H446. Therefore, Derlin-1 protein expression was high in non-small lung cancer cells.

### Changes in Derlin-1 expression by tunicamycin (TM)

**Figure 2:**
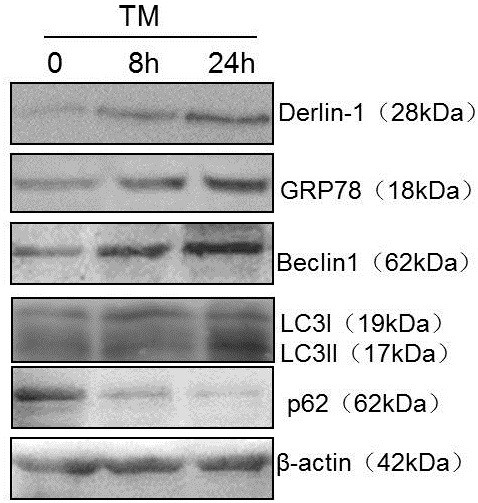
**Detection of Derlin-1 expression in NCI-N446 cells after TM treatment using western blot.** GRP78 expression was induced by ER stress. Expression of Derlin-1, autophagy gene Beclin 1 and LC3II increased under ER stress, but p62 expression decreased, indicating the induction of autophagy.

We detected Derlin-1 expression in NCI-H446 cells using ER stress-inducing agent TM. NCI-H446 cells were used because of their low endogenous level of Derlin-1. ER stress can be evoked by TM within a short time, so we detected gene expression 8 and 24 h after TM exposure. As an ER chaperone, glucose-regulated protein 78 (GRP78) is commonly used as a marker of ER stress. Increased GRP78 expression indicated that ER stress was triggered by TM treatment. Derlin-1 expression significantly increased after 8 and 24 h of TM treatment (Figure 2). In A549 cells with high endogenous levels of Derlin-1, Derlin-1 expression only slightly increased by TM treatment.

Meanwhile, we detected autophagy gene Beclin 1, LC3 and p62 expression under ER stress in NCI-H446 cells. As shown in Figure 2, we found that the expression of Beclin 1 and LC3II increased, but p62 expression decreased by TM treatment, which indicates that autophagy was triggered under ER stress. Monodansylcadaverine (MDC) is a special dye marker of autophagy that can be absorbed by cells and selectively combined with an autophagy bubble. Changes in the level of autophagy are demonstrated by fluorescence intensity. As shown in Additional file 1: Figure S1, the level of autophagy significantly increased after TM exposure in NCI-H446 cells. However, dots in the autophagy bubble were not obvious in this case. The same as in A549 cells (data not shown).

### Changes in autophagy gene expression after Derlin-1 siRNA transfection

**Figure 3:**
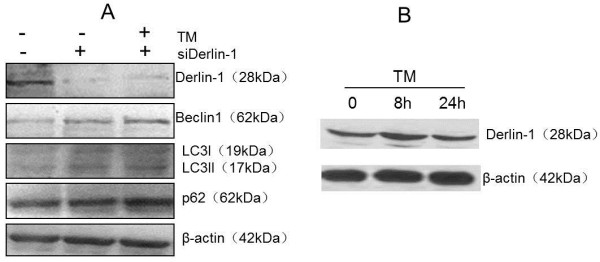
**Detection of Derlin-1 and autophagy marker gene expression after transfection of Derlin-1 siRNA with or without TM in A549 cells using western blot. A)** Detection of autophagy marker gene expression after transfection of Derlin-1 siRNA with or without TM in A549 cells. **B)** Detection of Derlin-1 expression after TM exposure in A549 cells. The results show no significant alteration in Beclin 1 and LC3II expression after Derlin-1 siRNA transfection, but the accumulation of p62 indicates a disruption in p62 degradation and impairment of autophagy flux.

To confirm the effect of Derlin-1 on autophagy gene expression, we transfected the siRNA target to Derlin-1 following TM treatment in A549 cells, which have high endogenous levels of Derlin-1. As shown in Figure 3, no significant alteration in Beclin 1 and LC3II expression was observed after Derlin-1 siRNA transfection. However, p62 accumulation was observed, indicating the disruption of p62 degradation and impairment of autophagy flux.

### Interaction between Derlin-1 and p62

**Figure 4:**
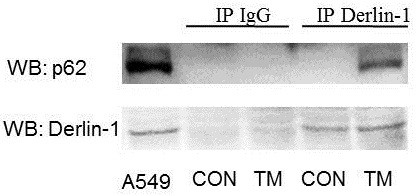
**Interplay of Derlin-1 and p62 in A549 cells under ER stress as demonstrated by co-immunoprecipitation assay.** Cells were treated with TM for 8 h. Derlin-1 antibody was used in immunoprecipitation. p62 and Derlin-1 antibody were used in western blot. The interaction between Derlin-1 and p62 was detected under ER stress.

We investigated the interplay between Derlin-1 and p62 under ER stress using co-immunoprecipitation assay in A549 cells. As shown in Figure 4, the interaction between Derlin-1 and p62 was detected under ER stress (Figure 4), but no detectable band of Beclin 1 and LC3II was observed under normal or ER stress conditions (data not shown).

## Discussion

Derlin-1 was initially reported to mediate the elimination of misfolded proteins from the ER and retrotranslocation of proteins into the cytosol [6]. Derlin-1 is a multifunctional protein. Its depletion in *Caenorhabditis elegans* results in ER stress [6]. Derlin-1 expression is upregulated by inducers of ER stress in yeast [14] and *C. elegans*[6]. Recent studies demonstrated the function of Derlin-1 in human cancers. A study using TMA showed that Derlin-1 is upregulated in six types of human carcinomas, and Derlin-1-targeting antibodies suppress colon tumour growth in isogenic mice [13]. Derlin-1 expression is elevated in breast and lung cancers, and correlated with tumour grade and lymph node metastasis [11,12]. Notably, Derlin-1 can relieve ER stress-induced apoptosis in breast cancer cells. In our study, we proved that the inhibition of autophagy led to aggravated ER stress, and induced downstream apoptosis in HeLa cells (to be published). We also aimed to determine whether Derlin-1, an ER-resident protein, participates in the regulation of autophagy to exert its oncogene function in tumour progression. Our findings reveal that knockdown of Derlin-1 could disrupt the degradation of p62 under ER stress. An interaction between Derlin-1 and p62 was detected using co-immunoprecipitation assay. This study is the first report about the correlation between Derlin-1 and p62. We also investigated the interplay between Derlin-1 and p62, as well as their effect on autophagy and other related signalling pathways.

First, we detected the expression of Derlin-1 in various lung cancer cells, including small lung cancer cells and non-small lung cancer cells. Our results show that the expression of Derlin-1 was higher in most non-small lung cancer cells than that in small lung cancer cells, especially in highly metastatic lung cancer cell line 95-D. This finding was in agreement with that of previous reports, which showed that Derlin-1 overexpression is associated with lymph node metastasis in human lung and breast cancers [11,12]. Anti-tumour drugs can induce ER stress in most cancer cells, and promote autophagic cell protection in almost all lung cancer cells [15]. Thus, we determined the expression of Derlin-1 in cells treated with lower levels of TM. TM increased Derlin-1 expression within a short period of time (8 h). Previous studies indicated that autophagy is induced under ER stress. We also observed the induction of autophagy by increased expression of Beclin 1, conversion of LC3I to LC3II and degradation of p62. Although the results show that the expression of these genes had been changed after TM treatment, whether Derlin-1 and autophagy are closely related remains inconclusive. We detected the expression of autophagy-related genes in A549 cells transfected with Derlin-1 siRNA to confirm the effect of Derlin-1 on autophagy. The expression levels of Beclin 1 and LC3II were not influenced by Derlin-1 siRNA transfection, but p62 accumulation was detected after Derlin-1 siRNA transfection. The interaction between Derlin-1 and p62 was initially verified using co-immunoprecipitation assay.

During the main pathway of autophagy, initially appearing membrane cisterns called phagophores capture portions of the cytoplasm in double-membrane autophagosomes. These vesicles then deliver cargo for lysosomal degradation [16,17]. Beclin 1, LC3II and p62 are considered to be the most important molecules during this process, and they are known as autophagy marker genes. p62 is a multifunctional signalling molecule involved in various cellular pathways. SQSTM1 is a well-known autophagic substrate that is widely used as an indicator of autophagic degradation. p62 facilitates selective autophagic removal of ubiquitinated cargo by binding to both ubiquitin and Atg8 covalently bound to the phagophore and autophagosome membranes [3,18], suggesting that autophagy has an important function in the degradation of ubiquitinated proteins. Studies showed that Derlin-1 is part of a multi-protein complex that mediates the dislocation, ubiquitination and extraction of ERAD substrates from the ER membrane [19,20]. Flierman reported that MHC-I molecules are rapidly dislocated and ubiquitinated once captured by Derlin-1 [21]. Therefore, the functional overlap of p62 and Derlin-1 in the degradation of ubiquitinated proteins may indicate their potential links. Our study only preliminarily investigated the suspected link between p62 and Derlin-1, but the underlying complex mechanism needs further investigation.

## Conclusions

Our findings indicate higher expression levels of Derlin-1 in non-small lung cancer cells than those in small lung cancer cells. The expression of Derlin-1 and autophagy marker genes was altered by ER stress. Knockdown of Derlin-1 may impair the degradation of p62 under ER stress. The interaction between Derlin-1 and p62 was identified in this study. However, further studies are necessary for exploring the functional relationship between Derlin-1 and p62 to widen our knowledge about Derlin-1 as an oncogene.

## Material and methods

### Cell lines and cell culture

Calu-3, GLC-82 and LTEP-a-2 cell lines were from ATCC (Manassas, VA) and 95-D, A549, NCI-N460, NCI-H209 and NCI-H446 were from Shanghai Cell Bank of Chinese Academy of Science (Shanghai, China). All human lung cancer cell lines were grown in Dulbecco’s minimal essential medium or RPMI 1640 medium containing 10% fetal bovine serum and 50 units/mL penicillin and 50 μg/mL streptomycin sulfate. Cells were incubated at 37°C in a humidified atmosphere of 5% CO_2_.

#### Reagent

Tunicamycin (TM) was purchased from Sigma-Aldrich (St. Louis, MO, USA) and dissolved in Me_2_SO at concentrations of 2 mg/mL for storage at −20°C. To induce ER stress, cells were treated with 2 μg/mL TM for 8 and 24 hours. Cells were plated 24 hours before TM was added to the fresh medium.

### Western blot analysis

For cultured cells, cells were washed twice with PBS and lysed with cold RIPA lysis buffer containing protease inhibitors (PMSF [phenylmethylsulphonyl fluoride] 1 mmol/L and leupeptin 0.1 g/L). Cell lysates were collected from culture plates using a rubber policeman, and protein was collected by centrifugation. Protein concentrations were determined by BCA (bicinchoninic acid) protein assay (Pierce, Rockford, IL, USA). Aliquots of 40 μg of proteins were boiled in 2× loading buffer (0.1 M Tris-Cl, pH 6.8, 4% SDS, 0.2% bromophenyl blue, and 20% glycerol) for 10 minutes, loaded into 10% Tris–HCl polyacrylamide gels, and transferred electrophoretically to Immobilon-P membrane (Millipore Corporation, Billerica, MA, USA). Membranes were incubated with primary antibodies and appropriate HRP secondary antibodies. Membranes were additionally probed with an antibody against β-actin to ensure equal loading of protein between samples. Detection was performed with chemiluminescent agents (Pierce). Derlin-1 (N-20, sc-46913), Beclin1 (H-300, sc-11427), GRP78 (H-129, sc-13968) and β-actin (C4, sc-47778) antibodies were from Santa Cruz Biotechnology, p62 (D5E2, 8025S), and LC3A/B (4108 s) antibodies were from Cell Signaling Technology.

### RNA interference

siRNA for derlin-1 was purchased from Santa Cruz Biotechnology (h, sc-60519), which contains a mixture of three siRNAs. Subconfluent proliferating cells in 6-well plates were incubated with 50 nM siRNA in 2 mL of medium containing Lipofectamine 2000 (Invitrogen Corporation) according to the manufacturer’s protocol.

### Immunoprecipitation assay

Cells were lysed by lysis buffer (50 mM Tris, pH 7.5, 150 mM NaCl, 1 mM NaF, 2 mM NaVO4, 1 mM EDTA, 1% Triton X-100, and protease inhibitor cocktail [Roche]) and sonicated. Cell lysates were centrifuged at 13000 rpm for 15 min at 4°C. The supernatant was incubated with antibodies for 2 h, then 50% A + G agarose beads was added for incubation for 1 h at 4°C. The precipitates were washed 3 times with lysis buffer for western blot analysis.

## Competing interests

The all authors declare no conflicts of any interests.

## Authors’ contributions

LX and ZHW conceived of the study, and participated in its design and coordination and helped to draft the manuscript, they contribute equally to this paper. DX carried out the protein expression experiments, and drafted the manuscript. GL carried out the co-immunoprecipitation assay. DRL and TW participated in the cell culture and siRNA transfection. SLG participated in the design of the study and performed the analysis. All authors read and approved the final manuscript.

## Authors’ information

Li Xu and Zan-Hong Wang co-first author.

## Supplementary Material

Additional file 1: Figure S1 Detection of autophagy by MDC staining in NCI-H446 cells without or with TM treatment for 8 h. The results show that levels of autophagy significantly increased after TM exposure. Strong fluorescence was observed in autophagy cells, whereas weak fluorescence was observed in non-autophagy cells. Click here for file
