# Supplementary figures and images for: Expression of Derlin-1 and its effect on expression of autophagy marker genes under endoplasmic reticulum stress in lung cancer cells
Source: Cancer Cell Int. 2014 Jun 11;14:50. doi: 10.1186/1475-2867-14-50 (PMC4061450; doi:10.1186/1475-2867-14-50)

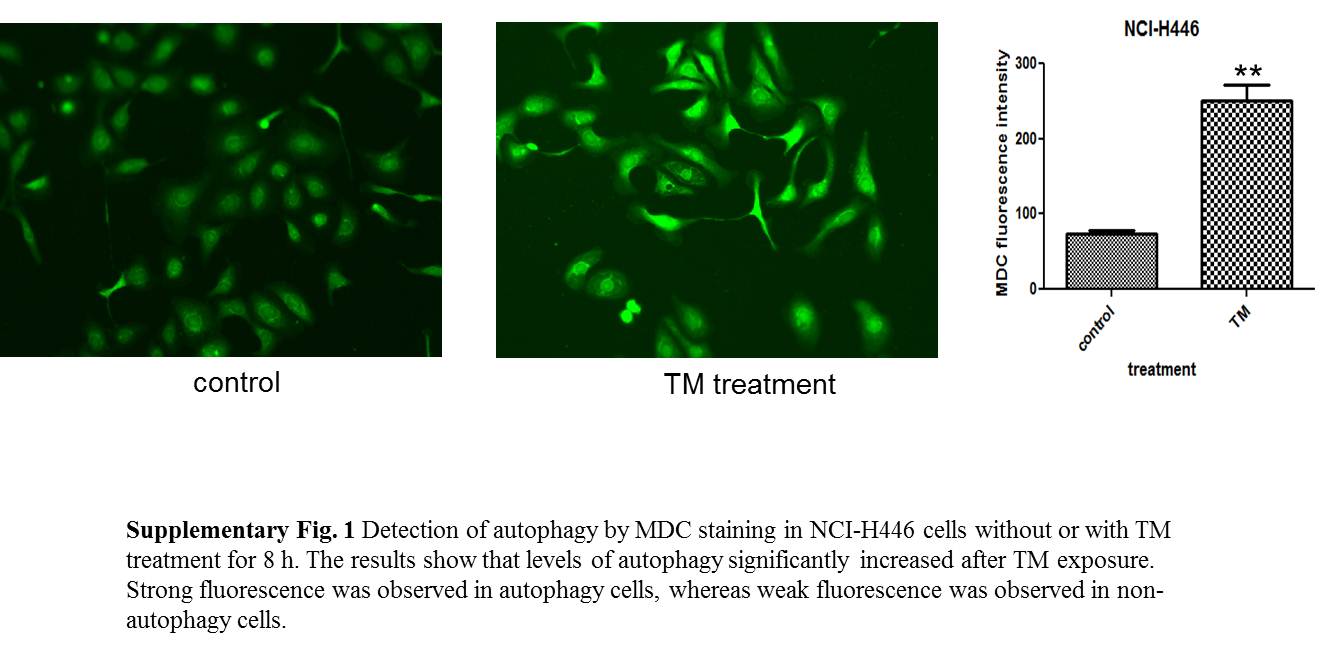

Supplement: Additional file 1: Figure S1 — Detection of autophagy by MDC staining in NCI-H446 cells without or with TM treatment for 8 h. The results show that levels of autophagy significantly increased after TM exposure. Strong fluorescence was observed in autophagy cells, whereas weak fluorescence was observed in non-autophagy cells. [file 1475-2867-14-50-S1.jpeg]
